# Acknowledgment to the Reviewers of *Nursing Reports* in 2022

**DOI:** 10.3390/nursrep13010012

**Published:** 2023-01-20

**Authors:** Nursing Reports Editorial Office

**Affiliations:** MDPI AG, St. Alban-Anlage 66, 4052 Basel, Switzerland

High-quality academic publishing is built on rigorous peer review. *Nursing Reports* was able to uphold its high standards for published papers due to the outstanding efforts of our reviewers. Thanks to the efforts of our reviewers in 2022, the median time to first decision was 22.5 days and the median time to publication was 48 days. Regardless of whether the articles they examined were ultimately published, the editors would like to express their appreciation and thank the following reviewers for the time and dedication that they have shown *Nursing Reports*:
Aaron, SiobhanLevitt, Cheryle G.Ahn, YongchelLi, WeixinAkın, BelginLima, AndreiaAlves, PauloLin, I-HsuanAminizadeh, MohsenLindegger, Daniel JosefAnnamária, Habil PakaiLis, AdrianaBecerra, ÁngelLopes-Júnior, Luís CarlosBhatta, DependraLorber, MatejaBobrova, Yulia O.Lucas, IsabelBorneman, TamiMacedo, João CarlosBretzke, JamesMacPhee, MauraCamedda, ClaudiaMagalhães, CátiaCangelosi, GiovanniMahta, AliCardinali, PaolaMajchrowska, AnitaCaruso, RosarioMarra, AnnachiaraCasal Angulo, Maria Del CarmenMartín-Conty, José LuisCasaña-Mohedo, JorgeMartínez, Juan PedroChang, SungokMartinez-Sabater, AntonioChavan, PrachiMatić, IvicaChen, Liang-ChingMattsson, JanetChen, SuiyaoMcClay, RebeccaCheng, Winnie L.S.McCormack, BrendanChenoweth, LynnMcFarlane, Shelly R.Chernet, AfonaMendes, José ManuelChow, Peggy Pik KeiMerino Godoy, Ma De Los ÁngelesCiarambino, TizianaMikaszewska-Sokolewicz, MałgorzataClapp, AlisonMonaco, Maria Grazia LourdesCoelho, AdrianaMoroń, MarcinConfalonieri, MarcoMoroni, MatteoConti, AlessioMukuni, JosephCordeiro, Raul Alberto CarrilhoMuleme, MichaelCorica, BernadetteNaim, SigalCosta, EmiliaNash, CarolDaddow, AngelaNowacki, KrzysztofDarbyshire, PamaylaNussbaum, DavidDo Arco, Helena ReisOh, JinaDobre, ClaudiaO’Hern, SteveDomagała, Przemysław MateuszOliveira, Isabel De JesusDoñate-Martínez, AscensiónOrtega-Campos, ElenaEchevarría-Pérez, PalomaOskarbski, JacekEdoh, Thierry OscarOwan, Valentine JosephFernandes, Júlio BeloOzga, DorotaFerri, LorenzoParola, VitorFino, EditaPereira, AlexandraForeman, Stephen E.Peritogiannis, VaiosGallego-Gómez, Juana InésPoddighe, DimitriGarau, CeliaPolak, IwonaGarcía-Mendoza, María Del CarmenPopoola, TosinGarcia-Moro, Francisco JoseQiu, FangfangGavin, KaraReig-Garcia, GlòriaGigol, TomaszRifkin, Susan B.Graveto, João Manuel Garcia Do NascimentoSabino, AnaGress- Halasz, BeataSancho-Cantus, DavidHall, Mellisa A.Santos-Jaen, Jose ManuelHalperin, OfraSegura, OriettaHamadi, Hanadi YShapira-Galitz, YaelHeczkova, JanaSheikhattari, PayamHemingway, SteveSilva, Rosa CarlaHemphill, Jean CroceSolís García Del Pozo, JuliánHernantes, NaiaSousa, Joana Sofia Dias Pereira DeHerrera-Peco, IvánSouza, Marcio Costa DeHertling, StefanStrizhitskaya, OlgaHiggins, NiallSzabo, GyulaHillman, HeidiTakase, KensakuHlaváčková, EvaTawia, SusanHuerta-González, SaraTreslova, MarieHutami, Islamy RahmaTsalta-Mladenov, Mihael EmilovHwu, Yueh-JuenTselebis, AthanasiosIsabel, Maria Tarico BicoTuclea, ClaudiaJankovic, MomciloTzeng, Huey-MingJiang, ChangchuanUchmanowicz, IzabellaJiménez Lasserrotte, María del MarUgarte Gurrutxaga, María IdoiaJosé, HelenaVan Cleeff, MaartenJulian-Rochina, IvanVasuki, RajaguruKamińska, Magdalena SylwiaVázquez, AndreaKim, Sang HunVelander, FredrikKim, Son ChaeVelarde-Garcia, Juan FranciscoKisielewska, JolantaVitale, ElsaKobylarek, AleksanderWalters, JaymeKoster, AndriesWan, Thomas T. H.Kotzampassi, KaterinaWinston, BruceKurnat-Thoma, EmmaWolfshaut-Wolak, RenataKuusisto, AnneWysokinski, MariuszLebel, UdiYang, Po-ChinLesińska-Sawicka, MałgorzataZarantonello, Francesco


